# Behavioral Intention of Receiving Monkeypox Vaccination and Undergoing Monkeypox Testing and the Associated Factors Among Young Men Who Have Sex With Men in China: Large Cross-Sectional Study

**DOI:** 10.2196/47165

**Published:** 2024-03-19

**Authors:** Sitong Luo, Kedi Jiao, Yuhang Zhang, Yutong Xu, Jingtao Zhou, Siwen Huang, Yan Li, Yongkang Xiao, Wei Ma, Lin He, Xianlong Ren, Zhen Dai, Jiaruo Sun, Qingyu Li, Feng Cheng, Wannian Liang

**Affiliations:** 1 Vanke School of Public Health Tsinghua University Beijing China; 2 Institute for Healthy China Tsinghua University Beijing China; 3 Guangdong Provincial Center for Disease Control and Prevention Guangzhou China; 4 Department of Acute Infectious Diseases Control and Prevention Anhui Provincial Center for Disease Control and Prevention Hefei China; 5 Department of Epidemiology School of Public Health Shandong University Jinan China; 6 Zhejiang Provincial Center for Disease Control and Prevention Hangzhou China; 7 Department of AIDS/STD Control and Prevention Beijing Center for Disease Control and Prevention Beijing China; 8 Department of AIDS/STD Control and Prevention Chengdu Center for Disease Control and Prevention Chengdu China

**Keywords:** mpox, monkeypox, young men who have sex with men, vaccination, testing, China, men, sex, cross-sectional study, infection, restrictions, feasibility, barrier, distress, emotional distress, men who have sex with men

## Abstract

**Background:**

The worldwide human monkeypox (mpox) outbreak in 2022 mainly affected men who have sex with men (MSM). In China, young men who have sex with men (YMSM) were at a potential high risk of mpox infection due to their sexual activeness and the eased COVID-19 restrictions at the end of 2022.

**Objective:**

This study aimed to investigate the behavioral intention of receiving mpox vaccination and undergoing mpox testing in 4 different scenarios and explore their associations with background and behavioral theory–related factors among Chinese YMSM.

**Methods:**

An online cross-sectional survey was conducted among YMSM aged 18-29 years from 6 representative provinces of China in September 2022. Participants recruited (recruitment rate=2918/4342, 67.2%) were asked to self-administer an anonymous questionnaire designed based on prior knowledge about mpox and classic health behavior theories. Data on the participants’ background, mpox knowledge and cognition, mpox vaccination and testing cognition, and the behavioral intention of receiving mpox vaccination and undergoing mpox testing were collected. Descriptive analysis and univariate and multivariate linear regressions were performed. Geodetector was used to measure the stratified heterogeneity of behavioral intention.

**Results:**

A total of 2493 YMSM with a mean age of 24.6 (SD 2.9) years were included. The prevalence of having a behavioral intention of receiving mpox vaccination ranged from 66.2% to 88.4% by scenario, varying in epidemic status and cost. The prevalence of having an mpox testing intention was above 90% in all scenarios regardless of the presence of symptoms and the cost. The positive factors related to vaccination intention included mpox knowledge (b_a_=0.060, 95% CI 0.016-0.103), perceived susceptibility of mpox (b_a_=0.091, 95% CI 0.035-0.146), perceived severity of mpox (b_a_=0.230, 95% CI 0.164-0.296), emotional distress caused by mpox (b_a_=0.270, 95% CI 0.160-0.380), perceived benefits of mpox vaccination (b_a_=0.455, 95% CI 0.411-0.498), self-efficacy of mpox vaccination (b_a_=0.586, 95% CI 0.504-0.668), and having 1 male sex partner (b_a_=0.452, 95% CI 0.098-0.806), while the negative factor was perceived barriers to vaccination (b_a_=–0.056, 95% CI –0.090 to –0.022). The positive factors related to testing intention were perceived severity of mpox (b_a_=0.283, 95% CI 0.241-0.325), perceived benefits of mpox testing (b_a_=0.679, 95% CI 0.636-0.721), self-efficacy of mpox testing (b_a_=0.195, 95% CI 0.146-0.245), having 1 male sex partner (b_a_=0.290, 95% CI 0.070-0.510), and having in-person gatherings with MSM (b_a_=0.219, 95% CI 0.072-0.366), while the negative factor was emotional distress caused by mpox (b_a_=–0.069, 95% CI –0.137 to –0.001).

**Conclusions:**

Among Chinese YMSM, the intention of undergoing mpox testing is optimal, while the mpox vaccination intention has room for improvement. A future national response should raise YMSM’s mpox knowledge, disseminate updated information about mpox and preventive measures, improve preventive service accessibility and privacy, and provide advice on positively coping with the associated emotional distress.

## Introduction

Since May 2022, rapidly growing numbers of human monkeypox (mpox) cases have been reported from countries where the disease was not initially endemic [1,2]. The World Health Organization (WHO) declared the worldwide mpox outbreak a public health emergency of international concern on July 23 [3]. By September 28, 2023, a total of 90,618 confirmed cases were reported across 115 countries, with 1484 confirmed cases in China. Among cases with known data on sexual orientation, 83.2% patients were identified as men who have sex with men (MSM) worldwide [4]. In China, more than 90% cases were identified as MSM [5]. One potential reason for MSM being the most affected population is that mpox transmission can result from close contact with the skin lesions of an infected person [1] and MSM communities usually have highly interconnected sexual networks [6].

China has the largest MSM population in the world, with an estimated size of 8.3 million [7], and among them, approximately 1.9 million are young people attending universities [8]. The first mpox case in mainland China was reported on September 16, 2022, and the patient was an MSM. The risk of an mpox outbreak became a public concern due to the large MSM population and the eased COVID-19 restrictions (eg, travel restrictions, social distancing) [9]. WHO recommended that the state parties with recently imported cases of mpox implement a coordinated response with a priority focus on the most affected communities [10].

Case detection and targeted immunization are powerful measures to fight the mpox outbreak [10-12]. Timely mpox testing can reduce mpox transmission, avoid delays in mpox treatment, and decrease the cost of health care [11-15]. Vaccination, such as that against smallpox, has been demonstrated to be highly cost-effective in preventing mpox infection [16]. Several classic theories postulate factors causing health-related behaviors and the underlying mechanisms. For example, the knowledge attitude practice (KAP) model [17] argues that knowledge of a disease and the related preventive/risk behaviors influences an individual’s attitude toward the behaviors and, in turn, affects the behaviors. The Health Belief Model (HBM) [18] proposes that people are more likely to take preventative actions if they perceive the threat of a health risk to be serious, feel they are personally susceptible, and perceive fewer costs than benefits of engaging in it. The theory of planned behavior (TPB) [19] says that attitudes, social norms, and perceived control of behaviors are predictors of behavioral intention. According to these theories and previous studies on other sexually associated infectious diseases, several factors may affect the behavioral intention of receiving mpox vaccination and undergoing mpox testing among MSM, including general factors (eg, sociodemographic characteristics, risk behaviors, disease knowledge, perceptions of health behaviors) and MSM-specific factors (eg, stigma) [20,21]. Although a large body of literature has examined preventive behaviors of diseases associated with MSM (eg, HIV/AIDS, sexually transmitted diseases [STDs], human papillomavirus [HPV]), the situation of mpox might be different, as it is a newly emerging disease in MSM communities. To timely inform the mpox prevention and control strategies in China, a comprehensive investigation on the behavioral intention of adopting mpox prevention measures and associated factors is warranted.

This study aimed to investigate the levels of the behavioral intention of receiving mpox vaccination and undergoing mpox testing among Chinese young men who have sex with men (YMSM) aged 18-29 years and to explore the associated factors. This age group was selected as it became the population with a high risk of mpox infection, especially after COVID-19 travel restrictions were eased in China. YMSM are the most socially active subgroup within MSM communities [22], have relatively more opportunities to meet people from abroad due to study or business reasons, and have not received smallpox vaccination, as China ended this immunization program in 1981 [23]. The hypotheses of potential factors were guided by the KAP model [17], the HBM [18], and the TPB [19].

## Methods

### Study Design

**Figure 1:**
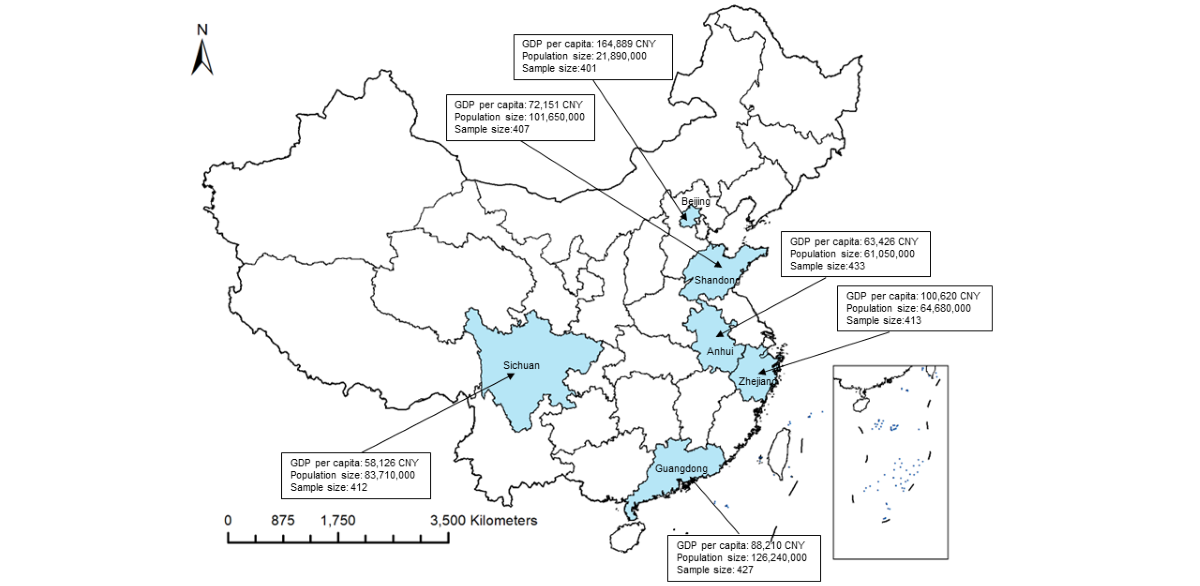
Map of the geographic location, per capita GDP, population size, and sample size of the 6 study sites in China. Note: The per capita GDP and population size of each study site were based on the data released by the National Bureau of Statistics of China in 2020. GDP: gross domestic product.

An online cross-sectional survey was conducted in September 2022 with participants from 6 provincial regions of China, which represented the country geographically and socioeconomically (based on the per capita gross domestic product [GDP] of 2020) to some extent [7,24]. These 6 regions were Beijing (the capital; per capita GDP=Chinese yuan [CNY] 164,889 [US $22,932]), Zhejiang (east; per capita GDP=CNY 100,620 [US $13,994]), Sichuan (west; per capita GDP=CNY 58,126 [US $8084]), Guangdong (south; per capita GDP=CNY 88,521 [US $12,311]), Shandong (north; per capita GDP=CNY 72,151 [US $10,034]), and Anhui (central; per capita GDP=CNY 63,426 [US $8821]). Figure 1 shows the geographic location, per capita GDP, population size, and sample size of the study areas.

### Participant Recruitment and Data Collection

The inclusion criteria were male participants, age 18-29 years, having ever had anal or oral sex with men, and ability to fill an electronic questionnaire and provide oral informed consent. The exclusion criteria were psychiatric disorders based on the recruiters’ report and refusal to participate.

The facility-based sampling method was used to recruit participants from each province with the assistance of local MSM-oriented community-based organizations (CBOs). The selected CBOs have rich experience in providing health services (eg, HIV/sexually transmitted infection [STI] testing) and conducting public health studies in MSM communities. First, CBO staff were trained as recruiters and asked to disseminate the invitation message to potentially eligible YMSM in their networks via commonly used messaging apps (eg, WeChat). YMSM who were interested in the study were then screened for eligibility and fully informed about the study. Next, they were sent a quick response (QR) code and a 1-time password to access the electronic anonymous questionnaire. The self-administered questionnaire took about 10-15 minutes to complete.

The recruitment rate of the study (number of returned questionnaires/number of invitations sent) was 67.2% (2918/4342). In each province, the recruitment rate ranged from 44.5% to 97.2%. The main reasons for nonparticipation (n=1424, 32.8%) reported by the recruiters were not meeting the inclusion criteria, lack of time, and privacy concerns. The 2918 participants completed the questionnaire, and 425 (14.6%) were excluded from analysis due to a failure in the quality check. The final sample size was 2493 (85.4%) participants.

### Ethical Considerations

The study was approved by the Institutional Review Board of Tsinghua University, China (reference number: 20220140). Each participant was asked to carefully read the informed consent form before starting the survey, in which the objective, procedure, potential risks and benefits, and voluntary nature of the study were clearly stated. Oral informed consent was obtained before any data collection. Written informed consent was waived to protect the participants’ privacy. The questionnaire was fully anonymous and self-administered by the participants through a secure privacy-compliant platform (Wenjuanxing survey platform [25]). Each participant whose submitted questionnaire passed the quality check receive Chinese yuan [CNY] 15 (about US $2.10) via the survey platform for compensation of their time and effort. To ensure confidentiality, further deidentified data were used for analysis.

### Measures

#### Behavioral Intention of Receiving Mpox Vaccination and Undergoing Mpox Testing

All the measures are described in detail in Multimedia Appendix 1. Regarding behavioral intention, the participants were asked to rate their likelihood (1=very unlikely to 5=very likely) of receiving mpox vaccination in 4 scenarios. A summative score was calculated, with a higher score indicating a higher level of intention (range 4-20, α=.90). Similarly, the participants were asked to rate their likelihood of undergoing an mpox test in 4 scenarios, with a higher summative score indicating a higher level of intention (range 4-20, α=.94).

#### Mpox Knowledge

Mpox knowledge was measured with a single yes/no question (“Have you heard of mpox?”) and 10 knowledge questions. A composite score of the knowledge scale was constructed by counting the number of correct responses to the questions; those who had not heard of mpox were assigned a score of 0 (score range 0-10). A higher score indicated a higher level of knowledge (α for the 10 knowledge questions=.77).

#### Mpox Cognition

The perceived susceptibility of mpox was measured with 3 items, with responses ranging from 1 for very unlikely to 5 for very likely. A higher total score implied a higher level of perceived susceptibility (range 3-15, α=.83). The perceived severity of mpox was measured with 2 items, with responses ranging from 1 for very mild to 5 for very strong. A higher summative score indicated a higher level of perceived severity (range 2-10, α=.90). Emotional distress (eg, panic, anxiety) caused by the mpox outbreak was measured with a single item, with the response ranging from 1 for very mild to 5 for very strong.

#### Cognition of Mpox Vaccination and Testing

Participants were asked about the perceived benefits, barriers, and self-efficacy of mpox vaccination/testing, with responses ranging from 1 for strongly disagree to 5 for strongly agree. The perceived benefits of mpox vaccination were assessed with 4 items (range 4-20, α=.90), and the perceived benefits of mpox testing were measured with 3 items (range 3-15, α=.91), with a higher summative score indicating more perceived benefits. The perceived barriers to mpox vaccination were measured with 4 items (range 4-20, α=.81), and the perceived barriers to mpox testing were measured with 5 items (range 5-25, α=.82), with a higher total score indicating more perceived barriers. The self-efficacy of mpox vaccination was measured with 2 items, with a higher summative score indicating a higher level of self-efficacy (range 2-10, α=.82). The self-efficacy of mpox testing was similarly measured (range 2-10, α=.85).

#### Background Information

Background information was collected, including (1) sociodemographic characteristics, (2) mpox risk behaviors in the past 6 months (eg, overseas travel history, number of male sex partners, frequency of in-person gatherings with MSM, use of any psychoactive substance before/during sexual intercourse [chemsex]), (3) diagnosis of HIV and STDs, and (4) presence of mpox-related symptoms in the past 2 weeks.

### Statistical Analysis

First, descriptive analyses were conducted, with means and SDs being reported for continuous variables and frequencies and percentages being reported for categorical variables. Second, the q-statistic Geodetector, a spatial variance analysis method that explains nonlinear associations, was used to measure and attribute the stratified heterogeneity [26]. The factor detector was applied to detect the spatial association between explanatory variables (ie, study sites, sociodemographic characteristics, and mpox risk behaviors) and response variables (ie, behavioral intention of receiving mpox vaccination and undergoing mpox testing). The q-statistic and *P* value of Geodetector were reported. Third, simple regression analyses were performed to explore the crude associations between the background/behavioral theory–related factors and behavioral intention. Fourth, multivariate linear regressions were performed, involving all the background and theoretical factors as independent variables. The point estimate and 95% CIs were reported. SPSS Statistics 24.0 (IBM Corporation) was used. Geodetector analysis was conducted using R version 4.3.1 (R Foundation for Statistical Computing).

## Results

### Background Characteristics

The participants’ mean age was 24.6 (SD 2.9) years. Most of the participants were Han Chinese (n=2392, 95.9%), employed (n=1755, 70.4%), and unmarried (n=2380, 95.5%); had completed college education or above (n=2086, 83.7%), reported a monthly income under CNY 6000 (US $836; n=1530, 61.3%), and self-identified as homosexual (n=2023, 81.1%). In the past 6 months, 51 (2%) reported an overseas travel history, 1336 (53.6%) had in-person gatherings with MSM for at least once, 1249 (50.6%) had more than 1 male sex partner, and 541 (21.7%) reported having had chemsex with MSM. A total of 2383 (95.6%) participants had ever undergone an HIV test, and 279 (11.2%) self-reported as HIV positive. In addition, 354 (14.2%) participants reported a history of STIs. Furthermore, 1923 (77.1%) participants reported they did not have any mpox-like symptoms in the past 2 weeks, and 113 (4.5%) reported they had close contact with people who showed mpox symptoms (Table 1).

**Table 1:** Distributions of background characteristics of YMSM^a^ from 6 provinces of China in September 2022 (N=2493).

Characteristics			Value
Age (years), mean (SD)			24.6 (2.9)
**Ethnicity, n (%)**			
	Han	2392 (95.9)	
	Others	101 (4.1)	
**Employment status, n (%)**			
	Student	611 (24.5)	
	Unemployed	127 (5.1)	
	Employed	1755 (70.4)	
**Education level, n (%)**			
	High school or below	407 (14.3)	
	College and above	2086 (83.7)	
**Monthly income (CNY^b^), n (%)**			
	≤2000 (US $279)	487 (19.5)	
	2001-6000 (US $279-$836)	1043 (41.8)	
	6001-10,000 (US $836-$1393)	652 (26.2)	
	>10,000 (US $1393)	311 (12.5)	
**Marital status, n (%)**			
	Married/living with a partner	113 (4.5)	
	Unmarried/divorced/separated/widowed	2380 (95.5)	
**Sexual orientation, n (%)**			
	Homosexual	2023 (81.1)	
	Bisexual	382 (15.3)	
	Heterosexual	8 (0.3)	
	Not sure/other	80 (3.2)	
**Overseas travel history, n (%)**			
	Yes	51 (2.0)	
	No	2442 (98.0)	
**Number of male sex partners, n (%)**			
	0	384 (15.5)	
	1	836 (33.9)	
	≥2	1249 (50.6)	
**Frequency of in-person gatherings with MSM^c^, n (%)**			
	Never	1157 (46.4)	
	1-2 times	710 (28.5)	
	3-5 times	332 (13.3)	
	1-3 times per month	223 (8.9)	
	1-4 times per week	59 (2.4)	
	>4 times per week	12 (0.5)	
**Having chemsex with MSM, n (%)**			
	Yes	541 (21.7)	
	No	1952 (78.3)	
**HIV infection, n (%)**			
	Positive	279 (11.2)	
	Negative/unknown/not clear	2104 (84.4)	
	Never tested	110 (4.4)	
**History of STIs^d^, n (%)**			
	Yes	354 (14.2)	
	No	2139 (85.8)	
**Having any mpox^e^symptoms, n (%)**			
	Yes	570 (22.9)	
	No	1923 (77.1)	
**Having close contact with people who showed mpox symptoms, n (%)**			
	Yes	113 (4.5)	
	No/not sure	2380 (95.5)	

^a^YMSM: young men who have sex with men.

^b^CNY: Chinese yuan. An exchange rate of CNY 1=US $0.14 has been applied.

^c^MSM: men who have sex with men.

^d^STI: sexually transmitted infection.

^e^mpox: monkeypox.

### Mpox Knowledge and Cognition

**Figure 2:**
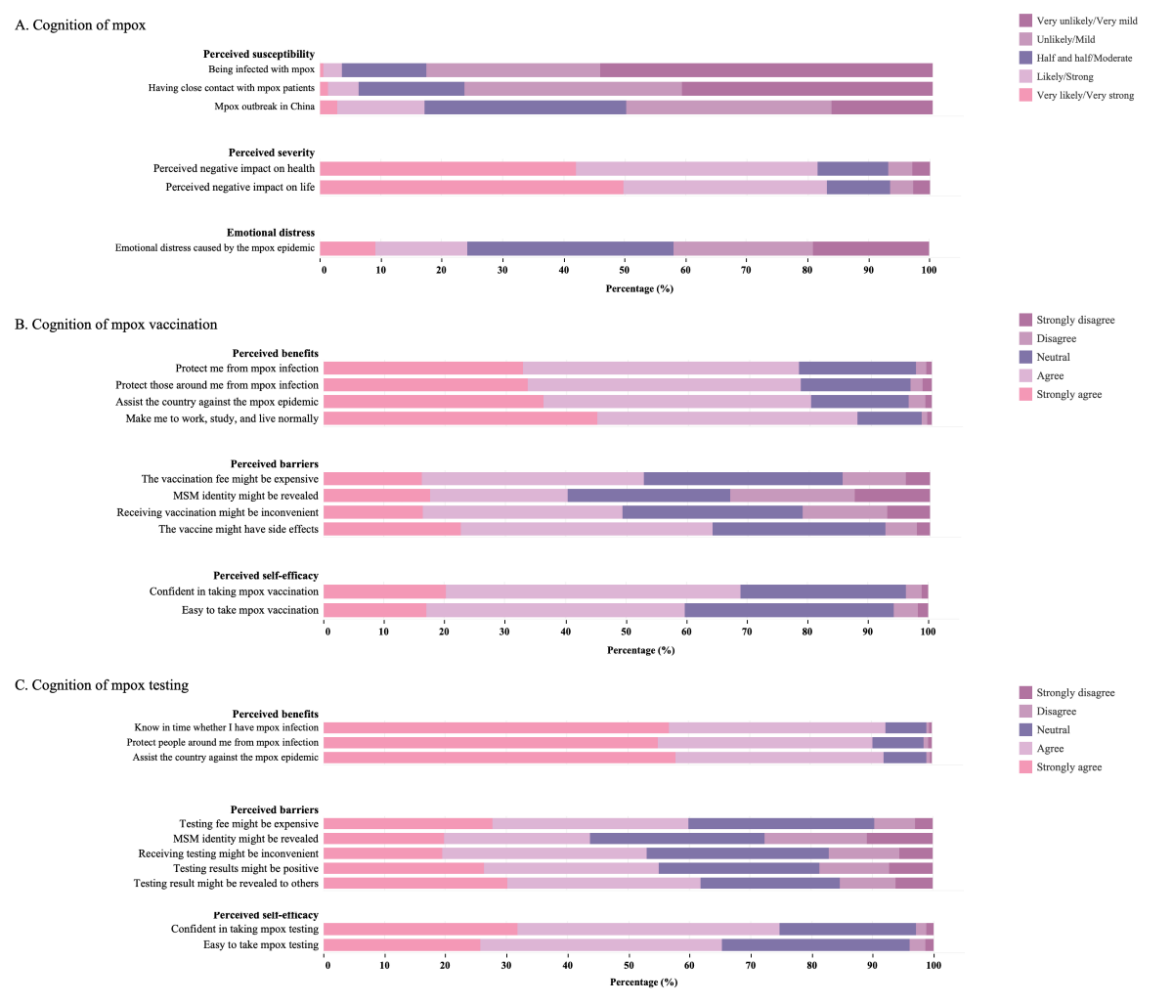
Level of cognition of mpox, mpox vaccination, and mpox testing among YMSM from 6 provinces in China in September 2022 (N=2493). mpox: monkeypox; YMSM: young men who have sex with men.

In total, 2310 (92.7%) participants had heard of mpox, and the proportions of choosing the correct answers for the 10 knowledge questions among the whole sample ranged from 13.4% (n=334) to 81.7% (n=2037), with a mean knowledge score of 5.7 (SD 2.7). Regarding perceived susceptibility (mean 6.1, SD 2.3), 91 (3.7%), 157 (6.3%), and 425 (17%) participants reported a high likelihood (likely or very likely) of being infected, of being in close contact with patients with mpox, and of an mpox outbreak in China, respectively. For perceived severity, 2035 (81.6%) and 2073 (83.2%) participants perceived a strong/very strong severity of mpox regarding their health and life, respectively (mean 8.4, SD 1.9). In addition, 605 (24.3%) participants reported being emotionally distressed due to mpox (mean 2.7, SD 1.2). See Table 2 and Figure 2.

**Table 2:** Distribution of mpox^a^-related knowledge and cognition of YMSM^b^ from 6 provinces of China in September 2022 (N=2493).

Variables			Value
Heard of mpox (frequency of choosing yes), n (%)			2310 (92.7)
**Mpox knowledge (frequency of choosing the right answer), n (%)**			
	Mpox virus can be transmitted to humans through close contact with an infected person.	2037 (81.7)	
	Mpox virus can be transmitted to humans through close contact with infected animals.	1860 (74.6)	
	Mpox virus can be transmitted to humans through close contact with contaminated objects.	1607 (64.5)	
	There are effective drugs against mpox virus in the world now.	1235 (49.5)	
	There are vaccines against mpox in China.	1241 (49.8)	
	After being infected with mpox virus, symptoms such as fever, rash, and swollen lymph nodes may occur.	1914 (76.8)	
	After a patient with mpox is cured, they may have scars for life once the skin rash goes away.	1535 (61.6)	
	Most patients with mpox can recover by themselves.	334 (13.4)	
	Symptoms are usually observed immediately once someone is infected with mpox.	1310 (52.5)	
	The smallpox vaccination can reduce the risk of mpox infection.	1141 (45.8)	
Total mpox knowledge score ranging from 0 to 10, mean (SD)			5.7 (2.7)
**Perceived susceptibility (frequency of choosing likely and very likely), n (%)**			
	Likelihood of being infected with mpox	91 (3.7)	
	Likelihood of having close contact with patients with mpox	157 (6.3)	
	Likelihood of an mpox outbreak in China	425 (17.0)	
Total perceived susceptibility score ranging from 3 to 15, mean (SD)			6.1 (2.3)
**Perceived severity (frequency of choosing strong and very strong), n (%)**			
	Perceived negative impact of mpox infection on your health	2035 (81.6)	
	Perceived negative impact of mpox infection on your life	2073 (83.2)	
Total perceived severity score ranging from 2 to 10, mean (SD)			8.4 (1.9)
**Emotional distress caused by mpox (frequency of choosing strong and very strong), n (%)**			
	Emotional distress caused by the mpox epidemic (eg, panic, anxiety)	605 (24.3)	
Total emotional distress score ranging from 1 to 5, mean (SD)			2.7 (1.2)

^a^mpox: monkeypox.

^b^YMSM: young men who have sex with men.

### Cognition of Mpox Vaccination and Testing

For the perceived benefits of mpox vaccination (mean 16.6, SD 2.9), the participants agreed/strongly agreed with the following 4 benefits presented: protecting oneself (n=1948, 78.1%), protecting others (n=1997, 80.1%), protecting the country (n=2187, 87.7%), and helping them live normally (n=1954, 78.4%). For the perceived barriers to mpox vaccination (mean 13.8, SD 3.5), the participants agreed/strongly agreed that the following 4 barriers are present: high cost of vaccination (n=1316, 52.8%), identity leakage (n=1002, 40.2%), inconvenience (n=1230, 49.3%), and side effects (n=1598, 64.1%). For self-efficacy (mean 7.5, SD 1.5), 1717 (68.9%) participants were confident in taking mpox vaccination and 1490 (59.8%) thought it would be easy to do so (Table 3 and Figure 2B).

**Table 3:** Behavioral intention and cognition related to mpox^a^ vaccination among YMSM^b^ from 6 provinces of China in September 2022 (N=2493).

Variables		Value
**Intention of receiving mpox vaccination by scenario** **(frequency of choosing likely and very likely), n (%)**		
	When there was no local case reported	1651 (66.2)
	When there was no local case reported and the vaccine was free	1971 (79.1)
	When there were local cases reported	2112 (84.7)
	When there were local cases reported and the vaccine was free	2204 (88.4)
Total intention score ranging from 4 to 20, mean (SD)		16.8 (3.6)
**Perceived benefits of mpox vaccination** **(frequency of choosing agree and strongly agree), n (%)**		
	It can protect me from mpox infection.	1948 (78.1)
	It can protect those around me from mpox infection.	1997 (80.1)
	It can assist the country in preventing and controlling the mpox epidemic.	2187 (87.7)
	It can make me to work, study, and live normally.	1954 (78.4)
Total perceived benefits score ranging from 4 to 20, mean (SD)		16.6 (2.9)
**Perceived barriers to mpox vaccination** **(frequency of choosing agree and strongly agree), n (%)**		
	I am worried that the vaccination fee might be high.	1316 (52.8)
	I am worried that my MSM^c^ identity might be revealed due to vaccination.	1002 (40.2)
	I am worried that receiving vaccination might be inconvenient.	1230 (49.3)
	I am worried that the vaccine might have side effects.	1598 (64.1)
Total perceived barriers score ranging from 4 to 20, mean (SD)		13.8 (3.5)
**Self-efficacy of mpox vaccination** **(frequency of choosing agree and strongly agree), n (%)**		
	I am confident in taking mpox vaccination.	1717 (68.9)
	I think it is easy for me to take mpox vaccination if I want.	1490 (59.8)
Total self-efficacy score ranging from 2 to 10, mean (SD)		7.5 (1.5)

^a^mpox: monkeypox.

^b^YMSM: young men who have sex with men.

^c^MSM: men who have sex with men.

For the perceived benefits of mpox testing (mean 13.4, SD 1.9), the participants agreed/strongly agreed with the following 3 benefits presented: timely finding out about the infection (n=2300, 92.3%), protecting others (n=2249, 90.2%), and protecting the country (n=2293, 92%). Regarding perceived barriers (mean 17.8, SD 4.4), the participants agreed/strongly agreed that the following 4 barriers are present: high cost (n=1495, 60%), inconvenience (n=1324, 53.1%), positive testing results (n=1370, 55%), identity leakage (n=1091, 43.8%), and leakage of testing results (n=1543, 61.9%). For self-efficacy (mean 7.9, SD 1.6), 1863 (74.7%) participants were confident in obtaining a mpox test and 1627 (65.3%) thought it would be easy to do so (Table 4 and Figure 2C).

**Table 4:** Behavioral intention and cognition related to mpox^a^ testing among YMSM^b^ from 6 provinces of China in September 2022 (N=2493).

Variables			Value
**Intention of getting mpox testing by scenario** **(frequency of choosing likely and very likely), n (%)**			
	When I had mpox symptoms	2302 (92.3)	
	When I had mpox symptoms and testing was free	2324 (93.2)	
	When I had close contact with patients with mpox	2320 (93.1)	
	When I had close contact with patients with mpox and testing was free	2342 (93.9)	
Total intention score ranging from 4 to 20, mean (SD)			18.5 (2.5)
**Perceived benefits of mpox testing** **(frequency of choosing agree and strongly agree), n (%)**			
	It can help me know in time whether I have an mpox infection.	2300 (92.3)	
	It can protect people around me from mpox infection.	2249 (90.2)	
	It can assist the country in preventing and managing mpox epidemics.	2293 (92.0)	
Total perceived benefits score ranging from 3 to 15, mean (SD)			13.4 (1.9)
**Perceived barriers to mpox testing** **(frequency of choosing agree and strongly agree), n (%)**			
	I am worried that the testing fee might be high.	1495 (60.0)	
	I am worried that my MSM^c^ identity might be revealed due to testing.	1091 (43.8)	
	I am worried that undergoing testing might be inconvenient.	1324 (53.1)	
	I am worried that my testing results might be positive.	1370 (55.0)	
	I am worried that my testing results might be revealed to others (eg, family members, colleagues, classmates).	1543 (61.9)	
Total perceived barriers score ranging from 5 to 25, mean (SD)			17.8 (4.4)
**Self-efficacy of mpox testing** **(frequency of choosing agree and strongly agree), n (%)**			
	I am confident in taking an mpox test.	1863 (74.7)	
	I think it is easy for me to take an mpox test if I want.	1627 (65.3)	
Total self-efficacy score ranging from 2 to 10, mean (SD)			7.9 (1.6)

^a^mpox: monkeypox.

^b^YMSM: young men who have sex with men.

^c^MSM: men who have sex with men.

### Behavioral Intention of Receiving Mpox Vaccination and Undergoing Mpox Testing

**Figure 3:**
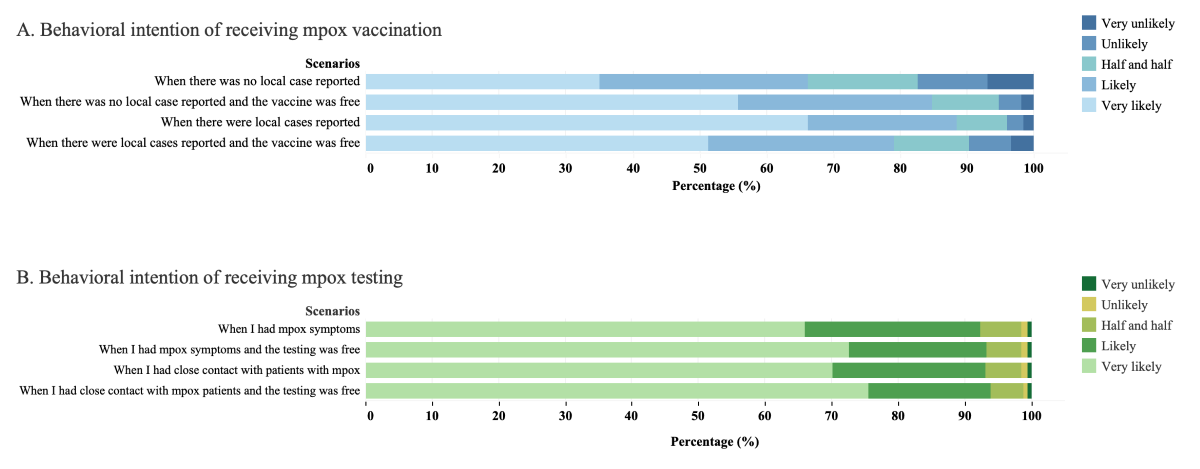
Behavioral intention of receiving mpox vaccination and testing in different scenarios among YMSM from 6 provinces of China in September 2022 (N=2493). mpox: monkeypox; YMSM: young men who have sex with men.

The prevalence of having a behavioral intention of receiving mpox vaccination varied in different scenarios (n=1651, 66.2%, when there was no local case reported; n=1971, 79.1%, when there was no local case reported and the vaccine was free; n=2112, 84.7%, when there were local cases reported; and n=2204, 88.4%, when there were local cases reported and the vaccine was free; mean 16.8, SD 3.6). See Table 3 and Figure 3A.

The prevalence of having a behavioral intention of undergoing mpox testing was above 90% in different scenarios (n=2302, 92.3%, when they had mpox symptoms; n=2324, 93.2%, when they had mpox symptoms and testing was free; n=2320, 93.1%, when they had close contact with patients with mpox; and n=2342, 93.9%, when they had close contact with patients with mpox and testing was free; mean 18.5, SD 2.5). See Table 4 and Figure 3B.

### Stratified Heterogeneity of Behavioral Intention of Receiving Mpox Vaccination and Undergoing Mpox Testing

As presented in Table 5, no heterogeneity was observed in the behavioral intention of receiving mpox vaccination and undergoing mpox testing across study sites and socioeconomic characteristics. However, heterogeneity was observed in the behavioral intention of receiving mpox vaccination in a number of male sexual partners (q=0.005, *P*=.04). In addition, heterogeneity was observed in the behavioral intention of undergoing mpox testing in in-person gatherings with MSM (q=0.002, *P*<.001).

**Table 5:** Geodetector analysis of driving factors of the behavioral intention of receiving mpox^a^ vaccination and undergoing mpox testing among YMSM^b^ from 6 provinces of China in September 2022 (N=2493).

Strata			Behavioral intention of receiving mpox vaccination (score)					Behavioral intention of undergoing mpox testing (score)		
		Mean (SD)		q-statistic (*P* value)^c^		Mean (SD)			q-statistic (*P* value)^c^	
**Study sites^d^**			—^e^		0.002 (.39)		—			0.003 (.14)
	Province A	16.79 (3.55)		—		18.72 (2.34)			—	
	Province B	16.93 (3.16)		—		18.51 (2.30)			—	
	Province C	16.88(3.50)		—		18.44 (2.30)			—	
	Province D	16.85 (3.70)		—		18.53 (2.58)			—	
	Province E	16.43 (3.70)		—		18.23 (2.73)			—	
	Province F	16.68 (3.89)		—		18.51 (2.67)			—	
**Age** **group (years)**			—		0.0006 (.26)		—			0.0002 (.47)
	18-24	16.85 (3.55)		—		18.45 (2.60)			—	
	25-29	16.68 (3.62)		—		18.53 (2.39)			—	
**Ethnicity**			—		0.0006 (>.99)		—			0.0006 (>.99)
	Han	16.74 (3.59)		—		18.48 (2.49)			—	
	Others	17.19 (3.49)		—		18.79 (2.54)			—	
**Employment status**			—		<0.0001 (.96)		—			0.001 (.76)
	Student/unemployed	16.75 (3.67)		—		18.38 (2.69)			—	
	Employed	16.77 (3.55)		—		18.54 (2.40)			—	
**Education level**			—		0.0002 (.96)		—			0.0002 (.99)
	High school or below	16.66 (3.71)		—		18.41 (2.84)			—	
	College and above	16.78 (3.57)		—		18.51 (2.42)			—	
**Monthly income (CNY^f^)**			—		0.0001 (.64)		—			0.004 (.61)
	≤6000 (≤US $836)	16.80 (3.56)		—		18.45 (2.54)			—	
	>6000 (>US $836)	16.71 (3.64		—		18.55 (2.41			—	
**Marital status**			—		<0.0001 (.99)		—			<0.0001 (.99)
	Married/living with a partner	16.63 (3.71)		—		18.45 (2.41)			—	
	Unmarried/divorced/separated/widowed	16.77 (3.58)		—		18.49 (2.49)			—	
**Overseas travel history**			—		<0.0001 (.99)		—			<0.0001 (>.99)
	Yes	16.84 (3.91)		—		18.71 (1.89)			—	
	No	16.76 (3.58)		—		18.49 (2.50)			—	
**Number of male sexual partners**			—		0.005 (.04)		—			0.008 (.07)
	0	16.16 (3.85)		—		17.98 (3.07)			—	
	1	16.89 (3.57)		—		18.68 (2.38)			—	
	≥2	16.88 (3.49)		—		18.52 (2.34)			—	
**In-person gatherings with MSM^g^**			—		0.001 (.08)		—			0.002 (.02)
	Yes	16.63 (3.79)		—		18.61 (2.25)			—	
	No	16.88 (3.41)		—		18.36 (2.74)			—	
**HIV infection**			—		0.004 (.50)		—			0.004 (.95)
	Positive	17.37 (3.39)		—		18.92 (2.19)			—	
	Negative/unknown/not clear/never tested	16.69 (3.61)		—		18.44 (2.52)			—	
**History of STIs^h^**			—		0.002 (.71)		—			0.002 (.98)
	Yes	17.14 (3.54)		—		18.74 (2.34)			—	
	No	16.70 (3.59)		—		18.45 (2.51)			—	
**Having chemsex with MSM**			—		0.002 (.38)		—			0.001 (.93)
	Yes	17.08 (3.43)		—		18.65 (2.31)			—	
	No	16.67 (3.63)		—		18.45 (2.54)			—	
**Having any mpox symptoms**			—		<0.0001 (.97)		—			0.002 (.81)
	Yes	16.79 (3.59)		—		18.69 (2.21)			—	
	No	16.75 (3.59)		—		18.43 (2.56)			—	
**Having close contact with people who showed mpox symptoms**			—		<0.0001 (.99)		—			0.0002 (>.99)
	Yes	16.70 (3.57)		—		18.33 (3.06)			—	
	No/not sure	16.77 (4.04)		—		18.50 (2.46)			—	

^a^mpox: monkeypox.

^b^YMSM: young men who have sex with men.

^c^The factor detector was used.

^d^Uppercase letters were used as pseudonyms of the study sites.

^e^Not applicable.

^f^CNY: Chinese yuan. An exchange rate of CNY 1=US $0.14 has been applied.

^g^MSM: men who have sex with men.

^h^STI: sexually transmitted infection.

### Factors Associated with the Behavioral Intention of Receiving Mpox Vaccination and Undergoing Mpox Testing

Table 6 presents the results of univariate and multivariate regression analyses of the behavioral intention of receiving mpox vaccination. The multivariate regression model showed that vaccination intention was significantly and positively associated with having 1 male sex partner in the past 6 months (b_a_=0.452, 95% CI 0.098-0.806), mpox knowledge (b_a_=0.060, 95% CI 0.016-0.103), perceived susceptibility (b_a_=0.091, 95% CI 0.035-0.146), perceived severity (b_a_=0.230, 95% CI 0.164-0.296), emotional distress (b_a_=0.270, 95% CI 0.160-0.380), perceived benefits (b_a_=0.455, 95% CI 0.411-0.498), and self-efficacy (b_a_=0.586, 95% CI 0.504-0.668). Perceived barriers were negatively associated with the vaccination intention (b_a_=–0.056, 95% CI –0.090 to –0.022).

**Table 6:** Univariate and multivariate regression analyses of the behavioral intention of receiving mpox^a^ vaccination among YMSM^b^ from 6 provinces of China in September 2022 (N=2493).

Independent variables			Univariate regression b_a_ (95% CI)		Multivariate regression b_a_ (95% CI)
Age (years)			–0.032 (–0.081 to 0.018)		–0.040 (–0.090 to 0.010)
Ethnicity (others vs Han)			0.443 (–0.272 to 1.158)		0.107 (–0.476 to 0.691)
Employment status (employed vs student/unemployed)			0.019 (–0.289 to 0.328)		0.028 (–0.295 to 0.352)
Education level (college and above vs high school or below)			0.119 (–0.262 to 0.500)		0.015 (–0.311 to 0.341)
Monthly income (>CNY^c^ 6000 vs ≤CNY 6000 [US $836])			–0.089 (–0.379 to 0.200)		–0.167 (–0.444 to 0.111)
Marital status (unmarried/divorced/separated/widowed vs married/living with a partner)			0.141 (–0.537 to 0.819)		–0.123 (–0.684 to 0.438)
Overseas travel history (yes vs no)			0.082 (–0.914 to 1.078)		0.216 (–0.593 to 1.024)
**Number of male sex partners in the past 6 months (reference: 0)**					
	1	0.730 (0.298 to 1.162)^d^		0.452 (0.098 to 0.806)^e^	
	≥2	0.726 (0.317 to 1.135)^d^		0.200 (–0.149 to 0.550)	
Having in-person gatherings in the past 6 months (yes vs no)			0.243 (–0.039 to 0.526)		0.058 (–0.179 to 0.295)
Having chemsex with male sex partners in the past 6 months (yes vs no)			0.409 (0.067 to 0.751)^e^		0.235 (–0.059 to 0.529)
HIV infection (yes vs other)			0.683 (0.236 to 1.129)^d^		0.321 (–0.066 to 0.708)
History of STIs^f^ (yes vs other)			0.434 (0.031 to 0.838)^e^		0.111 (–0.239 to 0.462)
Having any mpox symptoms in the past 2 weeks (yes vs no)			0.039 (–0.297 to 0.375)		–0.008 (–0.295 to 0.280)
Having close contact with people showing mpox symptoms in the past 2 weeks (yes vs other)			–0.067 (–0.744 to 0.611)		–0.191 (–0.762 to 0.381)
Mpox knowledge			0.156 (0.105 to 0.208)^g^		0.060 (0.016 to 0.103)^d^
Perceived susceptibility of mpox			0.183 (0.122 to 0.244)^g^		0.091 (0.035 to 0.146)^d^
Perceived severity of mpox			0.491 (0.417 to 0.565)^g^		0.230 (0.164 to 0.296)^g^
Emotional distress caused by mpox			0.571 (0.455 to 0.686)^g^		0.270 (0.160 to 0.380)^g^
Perceived benefits of mpox vaccination			0.622 (0.580 to 0.664)^g^		0.455 (0.411 to 0.498)^g^
Perceived barriers to mpox vaccination			0.051 (0.010 to 0.092)^e^		–0.056 (–0.090 to –0.022)^d^
Self-efficacy of receiving mpox vaccination			1.002 (0.919 to 1.085)^g^		0.586 (0.504 to 0.668)^g^

^a^mpox: monkeypox.

^b^YMSM: young men who have sex with men.

^c^CNY: Chinese yuan. An exchange rate of CNY 1=US $0.14 has been applied.

^d^*P*<.01.

^e^*P*<.05.

^f^STI: sexually transmitted infection.

^g^*P*<.001.

Table 7 presents the results of univariate and multivariate regression analyses of the behavioral intention of undergoing mpox testing. The testing intention was significantly and positively associated with having 1 male sex partner in the past 6 months (b_a_=0.290, 95% CI 0.070-0.510), having in-person gatherings with MSM (b_a_=0.219, 95% CI 0.072-0.366), perceived severity (b_a_=0.283, 95% CI 0.241-0.325), perceived benefits (b_a_=0.679, 95% CI 0.636-0.721), and self-efficacy (b_a_=0.195, 95% CI 0.146, 0.245). Emotional distress was negatively associated with testing intention (b_a_=–0.069, 95% CI –0.137 to –0.001).

**Table 7:** Univariate and multivariate regression analyses of the behavioral intention of undergoing mpox^a^ testing among YMSM^b^ from 6 provinces of China in September 2022 (N=2493).

Independent variables		Univariate regression b_a_ (95% CI)	Multivariate regression b_a_ (95% CI)
Age (years)		0.012 (–0.022 to 0.046)	–0.004 (–0.035 to 0.027)
Ethnicity (others vs Han)		0.313 (–0.183 to 0.809)	0.093 (–0.269 to 0.454)
Employment status (employed vs student/unemployed)		0.158 (–0.056 to 0.372)	0.141 (–0.059 to 0.341)
Education level (college and above vs high school or below)		0.103 (–0.161 to 0.368)	–0.105 (–0.307 to 0.097)
Monthly income (≤CNY^c^ 6000 vs >CNY 6000 [US $836])		0.102 (–0.099 to 0.303)	–0.100 (–0.272 to 0.071)
Marital status (unmarried/divorced/separated/widowed vs married/living with a partner)		0.042 (–0.428 to 0.513)	0.136 (–0.211 to 0.484)
Overseas travel history (yes vs no)		0.219 (–0.472 to 0.909)	0.498 (–0.002 to 0.998)
**Number of male sex partners in the past 6 months (reference: 0)**			
	1	0.700 (0.400 to 1.000)^d^	0.290 (0.070 to 0.510)^e^
	≥2	0.535 (0.251 to 0.818)^d^	0.061 (–0.156 to 0.278)
Having in-person gatherings in the past 6 months (yes vs no)		0.247 (0.051 to 0.443)^e^	0.219 (0.072 to 0.366)^f^
Having chemsex with male sex partners in the past 6 months (yes vs no)		0.208 (–0.029 to 0.445)	0.030 (–0.152 to 0.211)
HIV infection (yes vs other)		0.479 (0.170 to 0.789)^f^	0.140 (–0.099 to 0.380)
History of STIs^g^ (yes vs other)		0.293 (0.013 to 0.573)^e^	0.035 (–0.182 to 0.252)
Having any mpox symptoms in the past 2 weeks (yes vs no)		0.256 (0.024 to 0.489)^e^	0.140 (–0.038 to 0.317)
Having close contact with people showing mpox symptoms in the past 2 weeks (yes vs other)		–0.172 (–0.642 to 0.298)	–0.152 (–0.506 to 0.202)
Mpox knowledge		0.081 (0.045 to 0.117)^d^	–0.001 (–0.028 to 0.026)
Perceived susceptibility of mpox		–0.053 (–0.096 to –0.011)^e^	–0.024 (–0.059 to 0.011)
Perceived severity of mpox		0.531 (0.482 to 0.579)^d^	0.283 (0.241 to 0.325)^d^
Emotional distress caused by mpox		0.077 (–0.004 to 0.159)	–0.069 (–0.137 to –0.001)^e^
Perceived benefits of mpox vaccination		0.846 (0.808 to 0.884)^d^	0.679 (0.636 to 0.721)^d^
Perceived barriers to mpox vaccination		0.087 (0.065 to 0.109)^d^	0.010 (–0.007 to 0.027)
Self-efficacy of receiving mpox vaccination		0.597 (0.541 to 0.653)^d^	0.195 (0.146 to 0.245)^d^

^a^mpox: monkeypox.

^b^YMSM: young men who have sex with men.

^c^CNY: Chinese yuan. An exchange rate of CNY 1=US $0.14 has been applied.

^d^*P*<.001.

^e^*P*<.05.

^f^*P*<.01.

^g^STI: sexually transmitted infection.

## Discussion

### Principal Findings

This was among the earliest studies in China examining the levels and factors of the behavioral intention of receiving mpox vaccination and undergoing mpox testing among YMSM. After the COVID-19 travel restrictions were eased in China at the end of 2022, this specific age population became the group with a high risk of mpox infection due to its social activeness. The study findings shed some light on the preparedness for mpox prevention and control in the country.

### Comparison With Prior Work

Overall, about two-thirds of the surveyed YMSM intended to receive the mpox vaccination if there was no case present locally, and the prevalence increased to 84.7% if local cases were reported. The latter prevalence was higher than that in 2 other countries with an mpox outbreak. A recent study in the Netherlands showed that 70% of MSM (56.6% aged <45 years) had an intention of getting vaccinated [27]. Another study in France found that 33.6% of MSM (80.6% between 30 and 59 years old) declared their hesitancy to get vaccinated against mpox [28]. Age may be a potential reason for the relatively high prevalence, as YMSM tend to be more open to newly emerging health services (eg, pre-exposure prophylaxis [PrEP], HPV vaccines) [29-31]. The finding implies that it is important to keep MSM informed about the most up-to-date mpox statistics. It may assist them in decision-making about adopting preventive behaviors. Furthermore, the study found the prevalence of vaccination intention increased to some extent if the vaccination was free. This indicates that cost may be a barrier to mpox vaccination. The study showed about half of the participants expressed a concern about the potential high cost of vaccination, which is consistent with previous research [32]. Therefore, free or subsidized immunization for the high-risk population is recommended to be considered in the future national response.

In this study, over 90% of the participants reported an intention of undergoing an mpox test regardless of the presence of symptoms and the cost. A possible explanation for the high prevalence is that the participants may be knowledgeable about the benefits of testing. As shown in the study, most participants agreed/strongly agreed with the stated benefits of testing. The mass HIV testing campaigns in the past decades may have contributed to these perceptions. Our data showed that only 4.4% of the participants had never been tested for HIV before. Furthermore, the COVID-19 battle since 2020 may have improved people’s health literacy, which may, in turn, have positively affected their attitudes and practices of other health behaviors [33-35]. Lessons learned from the previous HIV testing campaigns targeting MSM and the COVID-19 testing campaign can be drawn by the future promotion of mpox testing in China [3,35]. For instance, it is imperative to remove structural barriers to testing (eg, provide anonymous tests) and offer culturally congruent services. Additionally, mpox testing is recommended to be provided in community settings, CBOs, and sexual health clinics trusted by MSM populations.

Guided by the classic health behavioral theories, we identified several factors associated with the behavioral intention of receiving mpox vaccination and undergoing mpox testing. First, according to the KAP model, knowledge is the foremost factor affecting the practice of preventive behaviors [17]. Previous studies have demonstrated that disease knowledge is positively associated with HIV testing and COVID-19–preventive behaviors [36,37]. Multivariate regression indicated a moderate association between mpox knowledge and vaccination intention and a statistically insignificant association between mpox knowledge and testing intention. The findings suggest that disease knowledge may play a role in motivating the intention of adopting preventive behaviors, but the strength and magnitude of the effect may be limited. Increasing mpox knowledge is important but far from enough in the future response to mpox in China.

The study found that 3 HBM constructs (perceived severity of mpox, perceived benefits of mpox vaccination/testing, and self-efficacy of mpox vaccination/testing) are independently and positively associated with vaccination and testing intentions. The findings are consistent with previous evidence on other diseases, such as COVID-19 and H1N1 [38,39]. Meanwhile, 2 other HBM constructs, perceived susceptibility of mpox and perceived barriers to the corresponding service, were also significantly associated with the vaccination intention but not with the testing intention. A possible reason is that the level of testing intention was particularly high in the study and the influence of the 2 factors were limited. Overall, these findings evidence that the HBM is a useful framework to predict health behaviors. Some related components are recommended for future mpox interventions, such as timely updating and disseminating mpox statistics, implementing targeted risk communication, educating the community about the benefits and ways of access to testing and vaccination, ensuring the privacy and confidentiality of services, and debunking and refuting misinformation promptly [40,41].

An interesting finding of this study was that the emotional distress caused by mpox is positively associated with the vaccination intention but negatively associated with the testing intention. It is understandable that YMSM who have experienced more emotional distress due to mpox might be more worried about mpox infection and thus intend to receive immunization. This is in line with previous research showing that people with more emotional distress caused by COVID-19 were more likely to receive COVID-19 vaccination [42]. In contrast, it is speculated that some YMSM were emotionally distressed because they were afraid that the testing result would be positive or the result would be revealed to others. Such fears consequently hindered their willingness to undergo testing. Our data showed that 55% of the participants were worried that their testing results might be positive, while 61.9% were worried that the testing results might be leaked to others. A previous HIV study also indicated that emotional distress is a major barrier to seeking HIV-testing services [43]. The opposite results remind us that it is necessary to monitor the mental status in the affected community during an emergency and provide timely advice on positive coping strategies.

Stratified heterogeneity analysis revealed variations in the behavioral intention among people with or without mpox risk behaviors. Certain mpox risk behaviors presented significant associations with the intention of receiving mpox vaccination and undergoing mpox testing. Those who had 1 male sex partner in the past 6 months were more intended to uptake both vaccination and testing services compared to those with none or multiple partners. It is understandable that MSM without sex partners recently might think they have no risk of infection and thus have no need to receive the vaccination or undergo testing, while MSM with multiple partners might possess a lower level of health literacy and risk perceptions [44]. The finding warns us that MSM with multiple sexual partners may have a lower intention of adopting preventive behaviors against mpox and should be given a priority focus in the future fight against mpox. In addition, attending in-person gatherings with MSM and traveling overseas in the past 6 months were both positively associated with the testing intention. The findings are expected because they are known risk factors of mpox infection [45,46].

It is worth noting that YMSM are at high risk of not only mpox but also HIV/AIDS. Evidence shows that mpox is an opportunistic infection of HIV/AIDS [47]. China has a well-established comprehensive public health system for HIV/AIDS health promotion, testing, treatment, and management, which is considered highly effective for HIV/AIDS high-risk groups. Therefore, it is highly recommended to integrate mpox-related health services and prevention actions into the existing HIV/AIDS prevention and control system. For example, it may be feasible and cost-effective to provide mpox vaccination and testing services at the current HIV testing and counseling sites that are often operated by MSM-friendly CBOs and clinics. It is also important to empower MSM communities and highly involve them, especially the key persons in the communities, in targeted health communication, health counseling, and testing and treatment referrals. This integrated approach is key to addressing the interconnected health challenges faced by YMSM.

### Limitations

The study has a few limitations. First, selection bias might exist due to the facility-based sampling and refusal to participate. YMSM who could be approached in this study might be more likely to interact positively with CBOs, receive health education information more frequently, and have a higher level of health literacy. Driven by self-selection, YMSM who were invited but refused to participate might tend to have more concerns about this sensitive topic and their privacy. These systematic difference biases potentially resulted in an overestimation of the behavioral intention related to mpox vaccination and testing. However, as MSM groups are hidden populations, the convenient sampling method is a common way for recruitment. In addition, data were collected from August to September 2022, when China was still under strict COVID-19 restrictions. The recruitment rate may have also been impacted by the pandemic. Second, due to self-reporting, social desirability bias might exist and lead to an overestimation of the behavioral intention. Third, the study was conducted in 6 provincial regions of China and focused on YMSM aged 18-29 years. The generalization of the study findings to broader populations should be made with caution. Fourth, the cross-sectional design allowed a limited ability of causal inferences. Longitudinal studies are warranted.

### Conclusion

The majority of the YMSM in this study showed an intention of receiving mpox vaccination, and the intention was somewhat sensitive to the epidemic status and cost. Most of the YMSM had an intention of undergoing an mpox test regardless of scenarios. It is recommended that the future national response to mpox in China prioritize groups with high-risk exposure, raise their mpox knowledge, promote cognition of the disease and preventive measures, improve accessibility and privacy of health services, monitor the mental health status of the groups, and provide advice on positive coping strategies.

## Appendix

Multimedia Appendix 1 Measures of the study.

## Abbreviations

CBO: community-based organization
CNY: Chinese Yuan
GDP: gross domestic product
HBM: Health Belief Model
HPV: human papillomavirus
KAP: knowledge attitude practice
mpox: monkeypox
MSM: men who have sex with men
STD: sexually transmitted disease
STI: sexually transmitted infection
TPB: theory of planned behavior
WHO: World Health Organization
YMSM: young men who have sex with men

## Data Availability

All data generated and analyzed during this study are included in this published paper and its supplementary information files.
