# Supplementary material for: Behavioral Intention of Receiving Monkeypox Vaccination and Undergoing Monkeypox Testing and the Associated Factors Among Young Men Who Have Sex With Men in China: Large Cross-Sectional Study
Source: JMIR Public Health Surveill. 2024 Mar 19;10:e47165. doi: 10.2196/47165 (PMC10988377; doi:10.2196/47165)
Supplement: Multimedia Appendix 1 [file publichealth_v10i1e47165_app1.docx]

**Appendix 1 Measures of the study**

| ***Mpox knowledge and cognitions*** |
| --- |
| **Mpox knowledge** |
| Have you heard of mpox? |
| Mpox virus can be transmitted to humans through close contact with an infected person |
| Mpox virus can be transmitted to humans through close contact with infected animals |
| Mpox virus can be transmitted to humans through close contact with contaminated objects |
| There are effective drugs against mpox virus in the world now |
| There are vaccines against mpox in China |
| After being infected with mpox virus, symptoms such as fever, rash, and swollen lymph nodes may occur |
| After the mpox patient being cured, they may have scar for life once the skin rash falls off |
| Most mpox patients can recover by themselves |
| The symptoms are usually observed immediately once someone is infected with mpox |
| The smallpox vaccination can reduce the risk of mpox infection |
| **Perceived susceptibility of mpox** |
| Likelihood of being infected with mpox |
| Likelihood of having close contact with mpox patients |
| Likelihood of mpox outbreak in China |
| **Perceived severity of mpox** |
| Perceived negative impact of mpox infection on your health |
| Perceived negative impact of mpox infection on your life |
| **Emotional distress caused by mpox** |
| Emotional distress caused by the mpox epidemic (e.g., panic, anxiety) |
| ***Mpox vaccination*** |
| **Intention of receiving mpox vaccination by scenarios** |
| When there was no local case reported |
| When there was no local case reported and the vaccine was free |
| When there were local cases reported |
| When there were local cases reported and the vaccine was free |
| **Perceived benefits of mpox vaccination** |
| It can protect me from mpox infection |
| It can protect those around me from mpox infection |
| It can assist the country in preventing and controlling the mpox epidemic |
| It can make me to work, study and live normally |
| **Perceived barriers to mpox vaccination** |
| I am worried that the vaccination fee might be expensive |
| I am worried that my MSM identity might be revealed due to vaccination |
| I am worried that receiving vaccination might be inconvenient |
| I am worried that the vaccine might have side effects |
| **Self-efficacy of mpox vaccination** |
| I am confident in taking mpox vaccination |
| I think it is easy for me to take mpox vaccination if I want |
| ***Mpox testing*** |
| **Intention of getting mpox testing by scenarios** |
| When I had mpox symptoms |
| When I had mpox symptoms and the testing was free |
| When I had close contact with mpox patients |
| When I had close contact with mpox patients and the testing was free |
| **Perceived benefits of mpox testing** |
| It can help me know in time whether I had mpox infection |
| It can protect people around me from mpox infection |
| It can assist the country in preventing and managing mpox epidemics |
| **Perceived barriers to mpox testing** |
| I am worried that the testing fee might be expensive |
| I am worried that my MSM identity might be revealed due to testing |
| I am worried that receiving testing might be inconvenient |
| I am worried that my testing results might be positive |
| I am worried that my testing result might be revealed to others (e.g., family members, colleagues, classmates) |
| **Self-efficacy of mpox testing** |
| I am confident in taking mpox testing |
| I think it is easy for me to take mpox testing if I want |
